# Feeding Algae Meal to Feedlot Lambs with Competent Reticular Groove Reflex Increases Omega-3 Fatty Acids in Meat

**DOI:** 10.3390/foods10020366

**Published:** 2021-02-08

**Authors:** Pilar Gómez-Cortés, Miguel Angel de la Fuente, Francisco Peña Blanco, Nieves Núñez-Sánchez, Francisco Requena Domenech, Andrés L. Martínez Marín

**Affiliations:** 1 Instituto de Investigación en Ciencias de la Alimentación (CIAL, CSIC-UAM), Universidad Autónoma de Madrid, Nicolás Cabrera, 9, 28049 Madrid, Spain; p.g.cortes@csic.es (P.G.-C.); mafl@if.csic.es (M.A.d.l.F.); 2 Departamento de Producción Animal, Universidad de Córdoba, Ctra. Madrid-Cádiz km 396, 14071 Córdoba, Spain; pa1peblf@uco.es (F.P.B.); pa2nusan@uco.es (N.N.-S.); 3 Departamento de Biología Celular, Fisiología e Inmunología, Universidad de Córdoba, Ctra. Madrid-Cádiz km 396, 14071 Córdoba, Spain; v02redof@uco.es

**Keywords:** functional food, intramuscular fat, EPA, DHA, DPA

## Abstract

The aim of this study was to compare the effects of supplementing marine algae as a source of omega-3 fatty acids (FA) in the diet, mixed in the concentrate or bottle-fed, on intramuscular fat FA composition of lambs with competent reticular groove reflex (RGR). Forty-eight feedlot lambs were distributed in three equal groups: one group did not consume marine algae nor had competent RGR, the second group received a daily dose (2.5%) of algae meal in the concentrate and the last group consumed the same dose of algae meal emulsified in milk replacer and bottle-fed. Marine algae raised the contents of EPA, DPA, and mainly DHA in the intramuscular fat, but the increase was significantly higher when algae meal was administered with a bottle via RGR. This strategy could contribute to improvements in the marketing of lamb meat by optimizing its status as a healthier food.

## 1. Introduction

The nutritional importance of the long chain omega-3 fatty acids (FA) EPA (eicosapentaenoic acid, 20:5), DPA (docosapentaenoic acid, 22:5) and DHA (docosahexaenoic acid, 22:6) in human nutrition is out of question, because of the long-term health benefits of their consumption, mainly prevention of cardiovascular diseases but also better visual and neurological development and improvements in chronic inflammatory conditions [1,2]. In the body, α-linolenic acid (ALA, 18:3), which is found in appreciable quantities in some vegetable foods such as walnuts and flaxseed, is the substrate for the synthesis of EPA and DHA; however, only a relatively small proportion of ALA is metabolized to EPA and even less to DHA [3]. Therefore, in order to obtain a regular supply of preformed EPA and DHA in the human diet, it should include foods from marine origin, which are naturally enriched in those FA [4].

Ovine meat is usually perceived as unhealthy due to its high levels of saturated FA (SFA) [5]. Moreover, intramuscular fat from lambs is usually poor in omega-3 FA [6]. Supplementing the diets of these animals with fat sources rich in omega-3 FA as fish oil [7], microalgae [8,9,10], and plant derived oils [9,11,12] has proven to be an appropriate strategy to obtain lamb meat enriched in omega-3. Thus, lamb meat would offer potential in terms of correcting omega-3 imbalances in human diet. However, the reported increases of omega-3 FA in meat or milk after the manipulation of ruminants’ diet are quantitatively low, mainly because the elevated biohydrogenation (BH) rates of dietary unsaturated FA in the rumen prevents a high absorption of them in the small intestine [13].

An alternative strategy to enhance the omega-3 FA content in ruminant derived foods could be to foster the reticular groove reflex (RGR) of the newborn animal into adulthood and use it to feed emulsified lipid sources into the abomasum, thus bypassing the rumen. This approach to protect dietary polyunsaturated fatty acids (PUFA) against rumen biohydrogenation was firstly hypothesized by Ørskov and Benzie [14]. Dobarganes García et al. [15] applied successfully the RGR in lactating ewes to bypass the rumen and change milk FA composition. More recently, Martínez Marín et al. [16] explored the RGR technique in adult lactating goats supplemented with emulsified flax oil and showed that it was possible to effectively bypass the rumen and, subsequently, modify the milk FA profile to obtain milk fat naturally rich in ALA. Early research by Lawlor et al. [17] demonstrated the feasibility of raising feedlot lambs on milk replacer plus concentrate, i.e., maintaining the RGR until slaughter weight (~34 kg), without adverse effects.

The need to increase omega-3 FA intake is related to reduction of cardiovascular disease risk, cognitive function improvement especially for an aging population, declining consumption of oily fish, and people relying on supplements for an adequate intake of EPA and DHA; whereas modification of animal fats through animal nutrition is feasible [18]. Previous studies have reported the effects of marine algae included in the solid feeds of the diet on intramuscular FA profile of lambs [10,19,20]. The aim of the current research was to investigate the usefulness of RGR to improve the levels of EPA and DHA in the meat of intensively reared feedlot lambs.

## 2. Materials and Methods

### 2.1. Experimental Design and Diets

This experiment was carried out in the premises of the Animal Production building of the University of Córdoba (Spain), in accordance with the Spanish regulations on protection of experimental animals (authorization no. 04/05/2018/074).

A total of 48 lambs from the Manchega breed were separated from their dams after feeding colostrum. From day one of age, they were bottle-fed milk replacer ad libitum, two times per day, up to 28 days of age. From 15 days of age onwards, a pelleted concentrate, based on cereals (70%) and soybean meal (20%), with no added fat, and formulated for growing lambs (16% crude protein and 11.0 MJ/kg metabolizable energy, as fed), was also offered as a means of stimulating rumen development. At 28 days of age, the daily volume of milk replacer was reduced to a fixed quantity of 500 mL, in a single feeding, in order to favor the intake of solid feeds. Seven days later, the daily volume of milk replacer was further reduced to 250 mL in a single feeding. The preparation of the milk replacer (Vigolait Active, Iniciativas Alimentarias S.A., Ciudad Real, Spain) was done according to the manufacturer’s directions.

At 42 days of age, lambs were weighed (11.6 ± 1.67 kg) and assigned to one of 24 pens, in pairs of similar body weight. The pens were 1.40-m^2^ raised slatted floor cages with individual troughs for feed and water. The cages were inside a closed room (84 m^2^) with windows (1:7 windows to floor ratio), a total of 16 h/d of natural plus artificial light (~1.5 W/m^2^ of artificial light), natural and forced ventilation, and controlled ambient temperature and humidity (20 to 25 °C and 60 to 80%, respectively; Poseidon2 3266, DITECOM, Madrid, Spain). The pens were blocked in 8 groups by their average body weight and were randomly allocated within each block to one of three treatments (16 animals per treatment), namely no added algae meal (NOALG), algae meal in concentrate (ALGCON), and algae meal in milk replacer (ALGMILK). In the NOALG treatment, the lambs did not longer receive milk replacer and were fed only the concentrate described above until slaughter. Lambs in the ALGCON treatment received a concentrate with the same ingredient composition as the NOALG treatment, but supplemented with 2.5% of algae meal plus 250 mL daily of milk replacer in a single feeding. The algae meal was added to the concentrate in the mixer before pelleting at the feed mill. The lambs in the ALGMILK treatment were fed the same concentrate as the NOALG treatment plus 250 mL daily of milk replacer supplemented with algae meal in a single feeding. The quantity of algae meal in the milk replacer was adjusted every three days to provide the same amount of lipid supplement as the average amount consumed by the lambs in the ALGCON treatment. The supplemented algae meal was Forplus^®^ (Alltech Spain, Guadalajara, Spain). Forplus^®^ is presented in powder form and according to the manufacturer is an unextracted, whole-cell microalgae (*Aurantiochytrium limacinum*) whose nutritional profile is 68.9% acid hydrolysis fat, 13.5% protein, 9.7% crude fiber, and 1.4% ash. The major FA composition of the ingredients in the experimental diets is shown in Table 1.

In all groups, the concentrate was offered in the morning every day to ensure at least 10% orts. Concentrate intake was calculated from feed offered and refused on a daily basis. Milk replacer was prepared every morning as described by Dobarganes García et al. [15] and bottle-fed just before offering the concentrate. Lambs had free access to wheat straw and fresh water throughout the experimental period.

### 2.2. Measurements, Sample Collection, and Laboratory Analyses

When the average body weight of the whole group of lambs reached ~25 kg (49 days on fattening), animals were transported in an adequately conditioned vehicle to a commercial abattoir. Lambs were stunned, slaughtered, and dressed using standard procedures. All carcasses were chilled at 4.0 °C for 24 h in a commercial chiller. Then, the carcasses were transferred to the laboratory without disrupting the cold chain. In the laboratory, samples of Longissimus muscle were obtained for intramuscular FA analysis after 6 days of ageing at 4 °C.

Intramuscular fat (IMF) of lamb meat samples was extracted using the Bligh and Dyer method [21] with a mixture of chloroform and methanol, adding BHT as antioxidant. Total lipids were preserved in amber vials and frozen at −20°C until their derivatization to fatty acid methyl esters (FAME). Acid-catalyzed methylation was carried out by reacting 25 mg of extracted muscle lipids with 1 mL of anhydrous HCl/CH3OH 5% w/v for 1 h at 80 °C according to Santercole et al. [22].

An Agilent model 6890 N Network System (Palo Alto, CA, USA) equipped with auto injector, fitted with an FID detector and a CP-Sil 88 fused silica capillary column (100 × 0.25 mm i.d., Varian, Middelburg, The Netherlands) was used for the lipid analyses. Injector and detector temperature was 250 °C. Helium was the carrier gas and inlet pressure was set at 194 kPa. The sample volume injected was 1 µL at a split ratio of 1:100. Total gas chromatographic (GC) time was 90 min. The initial oven temperature was 45 °C. After 4 min, it was raised at 13 °C min^–1^ to 165 °C and held for 35 min, then increased to 215 °C at 4 °C min^–1^ and maintained for 30 min. The identification of FAME was accomplished with GC standards acquired from Nu-Chek Prep Inc. (Elysian, MN, USA). Fatty acid quantification was carried out using 11:0 as internal standard.

### 2.3. Statistical Analysis

The MIXED procedure of SAS University Edition 3.8 (SAS Institute, Cary, NC, USA) was used for statistical analysis. The statistical model included the treatment as fixed effect and the pen nested within treatment as random effect. When the fixed effect was significant, differences between least squares means were assessed by Tukeys’s test. Statistical significance was declared at *p* < 0.05.

## 3. Results and Discussion

### 3.1. General Results and Fatty Acid Profile

No significant differences were found between experimental treatments on average feed intake (810 ± 41.1 g/d), average daily body weight gain (328 ± 29.0 g/d), or body weight at slaughter (25.4 ± 2.13 kg). The IMF content (2.2 ± 0.97%) was within the common range previously reported for Mediterranean light lambs reared indoors on concentrate-based diets [23,24]. It should be noted that the Bligh and Dyer method used to extract the IMF may underestimate meat total lipid content [25].

Overall IMF consisted of 37% to 40% SFA, 36% to 38% monounsaturated FA (MUFA), and 16% to 18% of PUFA (Table 2, Table 3 and Table 4). Palmitic (16:0), stearic (18:0), and oleic (*cis*-9 18:1) acids were the major individual FA in all treatments. This FA profile is within the range composition reported by Kaic et al. [6] from a meta-analysis of intramuscular FA contents in Mediterranean lambs.

### 3.2. Saturated Fatty Acids in Lamb Meat

The ALGCON and ALGMILK treatments promoted an IMF richer in total SFA (Table 2), mainly 16:0, which would reflect the composition of the diet, as palmitic acid was the most abundant FA in the algae meal (Table 1). These results are in agreement with Díaz et al. [10], who also found a higher proportion of 16:0 in the IMF of lambs fed algae meal in comparison with the control group. The desaturation indices of 14:0 (miristic acid) and 16:0 as well as the elongation index of 16 to 18 FA (Table 2) were higher in the NOALG treatment, which suggest that the activities of the stearoyl-CoA desaturases and elongases enzymes on the medium chain SFA [26] were inhibited in the algae meal supplemented lambs, probably due to an increased availability of PUFA in the tissues [9,27].

Several branched-chain FA from 15 to 18 atoms of carbon were detected, anteiso 17:0 being the most abundant (Table 2). Overall, the whole group of these FA represented less than 2% of total FA, but the content was lower in the algae meal supplemented treatments, likely due to the dilution effect created by the substantial increase of other FA groups. It is worth noting that despite their nutritional importance [28], branched-chain FA are less emphasized in the literature due to their scarcity in lamb meat [24].

### 3.3. Monounsaturated Fatty Acids in Lamb Meat

The contents of some minor *cis* MUFA showed differences between treatments, but total *cis* MUFA and the quantitatively main *cis* isomer, *cis*-9 18:1, were not affected by algae meal supplementation (Table 3). Regarding *trans* 18:1 FA, *trans*-10 was the major isomer in all meats as usually found in the IMF of lambs that are fed concentrate based diets, even those with a low starch proportion [24,29,30]. Interestingly, *trans*-10 18:1 content was lower in the NOALG and ALGMILK treatments than in the ALGCON treatment, which would suggest both, that the PUFA of algae meal boosted the *trans*-10 shifted rumen BH pathway in the ALGCON treatment [31] and that the RGR was effective in by-passing the rumen [16]. The second most important *trans* 18:1 isomer was *trans*-11 18:1 (vaccenic acid, VA) and was not modified by the experimental treatments.

### 3.4. Polyunsaturated Fatty Acids in Lamb Meat

The contents of some minor conjugated and non-conjugated 18:2 FA (e.g. *trans*-9 *cis*-11, *trans*-10 *cis*-12, *cis*-9 *cis*-15) showed higher values in the NOALG treatment than in the algae meal supplemented treatments, but total conjugated and non-conjugated 18:2 FA did not reach significant differences between treatments (Table 4). *Cis*-9 *trans*-11 18:2 (rumenic acid, RA) was not modified by algae meal supplementation, as previously observed by Díaz et al. [10] and Meale et al. [8], and its levels were below 0.1 % in all treatments. As pointed out by Bessa et al. [13], since most RA in ruminant tissues derive from delta-9 desaturation of VA, increasing RA contents in meat from intensively reared ruminants is usually constrained by the shift of ruminal BH pathways and the lower tissue availability of VA due to its replacement by *trans*-10 18:1.

Linoleic (*cis*-9 *cis*-12 18:2; LA) and arachidonic (*cis*-5 *cis*-8 *cis*-11 *cis*-14 20:4; AA) acids were the most abundant PUFA in all treatments (Table 4). The content of LA was not modified by lipid supplementation, but both algae meal supplemented treatments significantly decreased AA levels in IMF. It is noteworthy that the elongation index of LA to AA was higher in the NOALG when compared to the ALGCON and ALGMILK treatments (Table 4). It would be indicative of a higher activity of the delta-6 and delta-5 desaturase enzymes as well as of the ELOVl5 elongase enzyme [26] in the muscle cells of the lambs fed the unsupplemented diet. In this regard, Urrutia et al. [9] observed that the supplementation of lamb diets with long chain omega-3 PUFA downregulated the expression of delta-5 and delta-6 desaturase genes in muscle, showing a greater effect when the dietary supplement included algae meal. 

### 3.5. Omega-3 Fatty Acids in Lamb Meat

Table 4 shows the contents of the main omega-3 FA detected in the IMF of lambs fed the three experimental treatments. The ALA percentages were below 0.4% in all the diets assayed and without statistical differences between them. These results should be attributed to the limited presence of this omega-3 FA in the rations (Table 1). Lipid supplements of marine origin are poor in ALA and are not effective in increasing the levels of this omega-3 FA in ruminant products. However, adding linseed fat to the diet has demonstrated to be an effective way to increase ALA in lamb meat [9,12].

In comparison to ALA, DHA was exponentially risen when algae meal was introduced in the diets (Table 4). Marine algae are the primordial source of this long-chain omega-3 FA in the oceanic trophic chain. Therefore, the incorporation of microalgae in the diet has been considered an excellent way to increase DHA contents in ruminant derived foods [32]. The DHA content in the ALGCON treatment increased 19-fold in comparison with the NOALG treatment (Table 4) indicating that, despite ruminal BH, a high proportion of dietary DHA reached the small intestine intact, being absorbed and subsequently deposited in IMF. Cooper et al. [33] and Ponnampalam et al. [20] showed that dietary microalgae significantly enhanced DHA level in lamb muscle. In this regard, a 6.5-fold DHA increase was found when lambs were fed marine algae (2% DM of the ration) in comparison with the control diet [19]. Meale et al. [8] also reported 13 times more DHA in IMF from lambs fed with 3% DM of a microalgae.

In the current research, the highest DHA content in IMF (2.54% of total FA) was reached when algae meal was administered via bottle-fed. The DHA level in the ALGMILK IMF was 23 times higher than in the IMF of the NOALG treatment (Table 4). Thus, the RGR approach improved DHA levels by 20%, in comparison to conventional supplementation that incorporated algae meal in the concentrate. These results provide further evidence showing that it is possible to bypass the rumen effectively using the RGR strategy. Furthermore, it supports the potential of animals with competent RGR to successfully transfer dietary FA into ruminant-derived products as Dobarganes García et al. [15] and Martínez Marín et al. [16] previously reported.

The average algae meal intake was 20 g/d throughout the experimental period, which supplied approximately 4.6 g/d of DHA but only 0.07 g/d of EPA (Table 1). Despite the low intake of EPA, a significant increase was observed in the IMF from the ALGCON treatment with respect to the NOALG treatment, and such increment was even higher in the ALGMILK treatment (Table 4). These results could be explained by the retroconversion of DHA to EPA previously observed by Cooper et al. [33] and Díaz et al. [10] in lambs fed marine oil supplemented diets. Moreover, Alvarenga et al. [34] have observed that microalgae supplementation in lambs diet produced a higher expression of the FADS1 gene, which encodes delta-5 desaturase enzyme to catalyze the conversion of 20:4 omega-3 to EPA.

The results obtained in the present study have nutritional implications. One serving of 135 g of meat from the ALGCON and ALGMILK treatments would supply 88 and 135 mg of EPA + DHA, respectively, satisfying 35 and 43% of the daily recommended amounts (250 mg) by the European Food Safety Authority [35]. Furthermore, according to the European Union regulation [36] that defines a food as “source of omega-3 FA” if it contains at least 40 mg of EPA + DHA per 100 g and per 100 Kcal, the meat from the algae supplemented treatments could present such claim. Therefore, it is evident that the supplementation assayed in the present trial significantly increased omega-3 FA in lamb meat, contributing to daily EPA + DHA requirements, mainly when the incorporation was carried out via RGR.

Similarly to DHA and EPA, the highest contents of DPA in IMF were found in the ALGMILK treatment, whereas no significant differences were observed between the NOALG and ALGCON treatments (Table 4). Although no dietary recommendations have been made specifically for DPA and this omega-3 FA is not considered health-claimable [35,36], it may have unique beneficial effects in human nutrition. The DPA is precursor of anti-inflammatory and neuroprotective oxylipins, inhibits platelet aggregation more than DHA and EPA and is incorporated into phospholipids faster than EPA [37,38]. In fact, it has been suggested that DPA should be included in total omega-3 intake recommendations [39]. If so, the omega-3 provided by the meat from the ALGMILK treatment would raise to 153 mg per serving.

The ratio omega-6/omega-3 is generally considered to be essential when judging the nutritional value of foods. An important finding of the present research is that the addition of 2.5% of algae meal in feedlot lambs diet lowered the omega-6/omega-3 FA ratio from 11.3 (NOALG) to 3.2 and 2.7 in ALGCON and ALGMILK, respectively (Table 5). These values, in the range 2–3, are well below the threshold value of 4 that is considered adequate to prevent the pathogenesis of many diseases, including cardiovascular and chronic diseases [40]. Other quality indices are presented in Table 5. The differences between the control group and the algae meal supplemented treatments in the hypocholesterolemic/hypercholesterolemic ratio and the atherogenic index should be attributed to the higher consumption of preformed palmitic acid in the latter treatments (Table 1). Nevertheless, the values of those indices in all treatments were within the common ranges reported for meat in the literature [41]. The most raised peroxidizability index for the ALGMILK IMF would be a consequence of its high levels of pentanoic and hexanoic acids, which are more susceptible to oxidation.

## 4. Conclusions

Diet supplementation with marine algae significantly increased EPA, DPA, and DHA in lamb meat. In animals fed the marine algae in the concentrate, the omega-3 content of the IMF was enhanced to more closely resemble what is recommended for human consumption. Nevertheless, when marine algae was bottle-fed and bypassed the rumen thanks to the competent RGR, the improvement of the omega-3 profile in IMF was more effective. Appropriate feeding of marine algae to RGR competent ruminants would allow to obtain meat that might serve as a vehicle to enhance omega-3 FA intake in human diet, offering a suitable alternative to those who dislike oily fish. New studies in progress will provide information of the effects of this nutritional approach on chemical and instrumental quality traits related to sensory attributes of meat.

## Figures and Tables

**Table 1:** Fatty acid composition of the feeds used in the experimental treatments (g/100 g of total fatty acid methyl esters).

Fatty Acid	Control Concentrate	Algae Meal	Milk Replacer
8:0			1.36
10:0			1.34
12:0			16.23
14:0	0.26	7.17	5.84
15:0		1.52	
16:0	15.33	58.80	27.98
16:1		0.26	
17:0		0.38	
18:0	2.22	1.38	3.82
c9 18:1	22.91		30.48
18:2 omega-6 LA	51.64		11.12
18:3 omega-3 ALA	4.18		
20:0	0.44		
20:4 omega-6 AA		0.98	
20:5 omega-3 EPA		0.36	
22:0	0.23		
22:5 omega-6		3.24	
22:5 omega-3 DPA		0.32	
22:6 omega-3 DHA		22.66	
TOTAL SFA	18.48	69.51	56.57
TOTAL MUFA	22.91		30.48
TOTAL PUFA	55.82	27.56	11.12
TOTAL omega-3	4.18	23.34	
TOTAL omega-6	51.64	4.22	11.12

AA: arachidonic acid; ALA: α-linolenic acid; DHA: docosahexanoic acid; DPA: docosapentanoic acid; EPA: eicosapentanoic acid; LA: linoleic acid; MUFA: mononunsaturated fatty acids; PUFA: polyunsaturated fatty acids; SFA: saturated fatty acids.

**Table 2:** Saturated fatty acid (SFA) profile (g/100 g of total fatty acid methyl esters) in intramuscular fat of feedlot lambs fed a conventional diet alone (NOALG) or feedlot lambs with competent reticular groove reflex fed the same diet supplemented with 2.5% of algae meal, either mixed in the concentrate (ALGCON) or in the milk replacer (ALGMILK).

Fatty Acids	NOALG	ALGCON	ALGMILK	SEM	*p*
10:0	0.11 ^b^	0.15 ^a^	0.14 ^ab^	0.006	<0.05
12:0	0.08 ^b^	0.15 ^a^	0.17 ^a^	0.012	<0.01
13:0	0.02	0.02	0.02	0.001	0.87
14:0	1.31 ^b^	1.91 ^a^	1.81 ^a^	0.083	<0.01
15:0	0.42	0.36	0.37	0.019	0.36
16:0	16.51 ^b^	22.04 ^a^	20.87 ^a^	0.463	<0.001
17:0	2.41 ^a^	1.69 ^b^	1.71 ^b^	0.116	<0.05
18:0	13.88	12.32	13.36	0.266	0.06
20:0	0.08	0.07	0.08	0.002	0.20
21:0	0.05 ^a^	0.02 ^b^	0.02 ^b^	0.003	<0.001
22:0	0.11 ^a^	0.06 ^b^	0.06 ^b^	0.005	<0.001
23:0	0.04	0.03	0.04	0.006	0.48
24:0	0.05 ^c^	0.09 ^b^	0.11 ^a^	0.006	<0.001
TOTAL non-branched SFA	35.09 ^b^	38.89 ^a^	38.76 ^a^	0.490	<0.001
iso 15:0	0.05	0.05	0.05	0.002	0.69
anteiso 15:0	0.12	0.10	0.10	0.003	0.09
Other branched 15:0	0.12 ^a^	0.06 ^b^	0.06 ^b^	0.011	<0.05
iso 16:0	0.10	0.09	0.08	0.003	0.09
Other branched 16:0	0.47 ^a^	0.30 ^b^	0.31 ^b^	0.026	<0.05
iso 17:0	0.16 ^ab^	0.13 ^b^	0.17 ^a^	0.005	<0.01
anteiso 17:0	0.67 ^a^	0.51 ^b^	0.51 ^b^	0.017	<0.001
iso 18:0	0.16	0.14	0.13	0.007	0.17
TOTAL branched SFA	1.85 ^a^	1.39 ^b^	1.42 ^b^	0.056	<0.01
TOTAL SFA	36.94 ^b^	40.28 ^a^	40.18 ^a^	0.490	<0.01
14:0 desaturation index ^1^	0.04 ^a^	0.03 ^b^	0.03 ^b^	0.001	<0.001
16:0 desaturation index ^2^	0.06 ^a^	0.04 ^b^	0.04 ^b^	0.002	<0.001
18:0 desaturation index ^3^	0.65	0.68	0.66	0.005	0.07
16:0 elongation index ^4^	0.69 ^a^	0.62 ^b^	0.64 ^b^	0.007	<0.001

^1^ c9 14:1/(c9 14:1 + 14:0); ^2^ c9 16:1/(c9 16:1 + 16:0); ^3^ c9 18:1/(c9 18:1 + 18:0); ^4^ (18:0 + c9 18:1)/(16:0 + c9 16:1 + 18:0 + c9 18:1); ^abc^ Within the same row, least squares means without a common superscript letter differ significantly (*p* < 0.05) by Tukey’s test. SEM: standard error of the mean.

**Table 3:** Monounsaturated fatty acid (MUFA) profile (g/100 g of total fatty acid methyl esters) in intramuscular fat of feedlot lambs fed a conventional diet alone (NOALG) or feedlot lambs with competent reticular groove reflex fed the same diet supplemented with 2.5% of algae meal, either mixed in the concentrate (ALGCON) or in the milk replacer (ALGMILK).

Fatty Acids	NOALG	ALGCON	ALGMILK	SEM	*p*
c9 14:1	0.05	0.05	0.05	0.002	0.60
c7 16:1	0.18 ^a^	0.15 ^b^	0.16 ^ab^	0.005	<0.05
c9 16:1	1.00	0.98	0.84	0.033	0.10
c9 18:1 + (c10+t13+t14) 18:1	25.68	25.82	26.21	0.484	0.86
c11 18:1 + t15 18:1	3.20 ^a^	2.58 ^b^	2.66 ^b^	0.065	<0.001
c12 18:1	0.25 ^ab^	0.18 ^b^	0.30 ^a^	0.015	<0.01
c13 18:1	0.14 ^a^	0.06 ^b^	0.04 ^b^	0.007	<0.001
c16 18:1	0.02	0.02	0.02	0.002	0.60
20:1	0.03 ^a^	0.03 ^a^	0.02 ^b^	0.001	<0.001
c8 20:1	0.02 ^a^	0.01 ^b^	0.01 ^b^	0.001	<0.001
c11 20:1	0.13	0.12	0.10	0.006	0.08
22:1	0.04 ^c^	0.08 ^b^	0.11 ^a^	0.006	<0.001
c15 24:1	0.02	0.02	0.02	0.003	0.84
TOTAL *cis* MUFA	30.73	30.11	30.52	0.484	0.86
t8 16:1	0.08 ^a^	0.09 ^a^	0.06 ^b^	0.004	<0.001
t9 16:1	0.12	0.13	0.14	0.006	0.73
t5 18:1	0.02	0.02	0.02	0.001	0.16
t(6+7+8) 18:1	0.15 ^a^	0.05 ^b^	0.13 ^a^	0.011	<0.001
t10 18:1	3.94 ^b^	6.02 ^a^	2.71 ^b^	0.488	<0.001
t11 18:1	2.25	1.95	2.06	0.222	0.70
TOTAL *trans* MUFA	7.42 ^ab^	7.75 ^a^	6.08 ^b^	0.266	<0.05
TOTAL MUFA	38.15	37.86	36.60	0.592	0.50

c: *cis*; t: *trans*; ^abc^ Within the same row, least squares means without a common superscript letter differ significantly (*p* < 0.05) by Tukey’s test. SEM: standard error of the mean.

**Table 4:** Polyunsaturated fatty acid (PUFA) profile (g/100 g of total fatty acid methyl esters) in intramuscular fat of feedlot lambs fed a conventional diet alone (NOALG) or feedlot lambs with competent reticular groove reflex fed the same diet supplemented with 2.5% of algae meal, either mixed in the concentrate (ALGCON) or in the milk replacer (ALGMILK).

Fatty Acids	NOALG	ALGCON	ALGMILK	SEM	*p*
t9 t12 18:2	0.02 ^b^	0.03 ^a^	0.01^c^	0.001	<0.001
c9 t13 + t8 c12 18:2	0.06	0.05	0.07	0.004	0.14
c9 t12 + t8 c13 18:2	0.04	0.03	0.05	0.003	0.13
t9 c12 18:2	0.02	0.02	0.02	0.001	0.21
t11 c15 18:2	0.16 ^ab^	0.20 ^a^	0.12 ^b^	0.010	<0.01
c9 c12 18:2 LA	10.64	8.80	8.83	0.344	0.06
c9 c15 18:2	0.28 ^a^	0.19 ^b^	0.18 ^b^	0.010	<0.001
TOTAL non-conjugated 18:2	11.22	9.34	9.27	0.353	0.06
c9 t11 18:2 RA	0.09	0.09	0.10	0.022	0.93
t9 c11 18:2	0.09 ^a^	0.06 ^b^	0.05 ^b^	0.004	<0.001
t10 c12 18:2	0.06 ^a^	0.03 ^b^	0.02 ^b^	0.003	<0.001
TOTAL conjugated 18:2	0.24	0.17	0.27	0.022	0.11
TOTAL 18:2	11.46	8.93	9.54	0.402	0.06
18:3 omega-6	0.12 ^a^	0.06 ^b^	0.08 ^b^	0.005	<0.001
18:3 omega-3 ALA	0.36	0.32	0.32	0.009	0.24
20:2 omega-6	0.08	0.07	0.07	0.003	0.06
20:3 omega-3	0.33 ^a^	0.16 ^b^	0.18 ^b^	0.016	<0.001
20:3 omega-6	0.32 ^a^	0.25 ^b^	0.29 ^ab^	0.015	0.06
20:4 omega-6 AA	4.57 ^a^	2.82 ^b^	2.99 ^b^	0.242	<0.01
20:5 omega-3 EPA	0.20 ^c^	0.86 ^b^	1.27 ^a^	0.085	<0.001
22:3 omega-3	0.04	0.04	0.04	0.002	0.55
22:4 omega-6	0.56 ^a^	0.16 ^b^	0.13 ^b^	0.034	<0.001
22:5 omega-6	0.17 ^b^	0.25 ^a^	0.27 ^a^	0.014	<0.01
22:5 omega-3 DPA	0.42 ^b^	0.45 ^b^	0.62 ^a^	0.027	<0.01
22:6 omega-3 DHA	0.11^c^	2.12 ^b^	2.54 ^a^	0.182	<0.001
TOTAL PUFA	18.77	16.49	18.31	0.722	0.41
TOTAL omega-3	1.45^c^	3.94 ^b^	4.96 ^a^	0.274	<0.001
TOTAL omega-6	16.46 ^a^	12.41 ^b^	12.64 ^b^	0.626	<0.05
c9 c12 18:2 elongation index ^1^	0.29 ^a^	0.24 ^b^	0.25 ^b^	0.007	<0.01

^1^ 20:4 n-6/(c9 c12 18:2 + 20:4 n-6); c: *cis*; t: *trans*; ^abc^ Within the same row, least squares means without a common superscript letter differ significantly (*p* < 0.05) by Tukey’s test. AA: arachidonic acid; ALA: α-linolenic acid; DHA: docosahexanoic acid; DPA: docosapentanoic acid; EPA: eicosapentanoic acid; LA: linoleic acid; PUFA: polyunsaturated fatty acids; RA: rumenic acid; SEM: standard error of the mean.

**Table 5:** Lipid quality indices in intramuscular fat of feedlot lambs fed a conventional diet alone (NOALG) or feedlot lambs with competent reticular groove reflex fed the same diet supplemented with 2.5% of algae meal, either mixed in the concentrate (ALGCON) or in the milk replacer (ALGMILK).

Index	NOALG	ALGCON	ALGMILK	SEM	*p*
omega-6/omega-3	11.27 ^a^	3.16 ^b^	2.71 ^b^	0.581	<0.001
PUFA/SFA	0.53	0.41	0.46	0.024	0.15
h/H ^1^	2.63 ^a^	1.88 ^b^	2.06 ^b^	0.066	<0.001
AI ^2^	0.39 ^b^	0.56 ^a^	0.52 ^a^	0.018	<0.001
TI ^3^	1.00	0.98	0.92	0.023	0.22
PI ^4^	41.26 ^b^	49.83 ^ab^	57.99 ^a^	2.441	<0.01
DFA ^5^	70.70 ^a^	66.66 ^b^	68.27 ^ab^	0.499	<0.05

^1^ Hypocholesterolemic/hypercholesterolemic index = (c9 18:1 + c11 18:1 + 18:2 omega-6 + 18:3 omega-6 + 18:3 omega-3 + 20:3 omega-6 + 20:4 omega-6 + 20:5 omega-3 + 22:4 omega-6 + 22:5 omega-3 + 22:6 omega-3)/(14:0 + 16:0). ^2^ Atherogenic index = (12:0 + 4 × 14:0 + 16:0)/Σ UFA. ^3^ Thrombogenic index = (14:0 + 16:0 + 18:0)/[(0.5 × MUFA) + (0.5 × Σ omega-6) + (3 × Σ omega-3) + (Σ omega-3/Σ omega-6)]. ^4^ Peroxidizability index = (monoenoic acid × 0.025) + (dienoic acid × 1) + (trienoic acid × 2) + (tetraenoic acid × 4) + (pentaenoic acid × 6) + (hexaenoic acid × 8). ^5^ Desirable fatty acids = MUFA + PUFA + 18:0. MUFA: monounsaturated fatty acids; PUFA: polyunsaturated fatty acids; SFA: saturated fatty acids; UFA: unsaturated fatty acids. ^ab^ Within the same row, least squares means without a common superscript letter differ significantly (*p* < 0.05) by Tukey’s test. SEM: standard error of the mean.

## Data Availability

The data presented in this study are available in the article.

## References

[1] Calder P.C. (2018). Very long-chain n-3 fatty acids and human health: Fact, fiction and the future. Proc. Nutr. Soc..

[2] Shahidi F., Ambigaipalan P. (2018). Omega-3 Polyunsaturated Fatty Acids and Their Health Benefits. Annu. Rev. Food Sci. Technol..

[3] Baker E.J., Miles E.A., Burdge G.C., Yaqoob P., Calder P.C. (2016). Metabolism and functional effects of plant-derived omega-3 fatty acids in humans. Prog. Lipid Res..

[4] Cholewski M., Tomczykowa M., Tomczyk M. (2018). A comprehensive review of chemistry, sources and bioavailability of omega-3 fatty acids. Nutrients.

[5] Chikwanha O.C., Vahmani P., Muchenje V., Dugan M.E., Mapiye C. (2018). Nutritional enhancement of sheep meat fatty acid profile for human health and wellbeing. Food Res. Int..

[6] Kaic A., Mioc B., Kasap A., Potocnik K. (2016). Meta-analysis of intramuscular fatty acid composition of Mediterranean lambs. Arch. Anim. Breed..

[7] Ponnampalam E.N., Sinclair A.J., Egan A.R., Blakeley S.J., Li D., Leury B.J. (2001). Effect of dietary modification of muscle long-chain n-3 fatty acid on plasma insulin and lipid metabolites, carcass traits, and fat deposition in lambs. J. Anim. Sci..

[8] Meale S.J., Chaves A.V., He M.L., McAllister T.A. (2014). Dose-response of supplementing marine algae (*Schizochytrium* spp.) on production performance, fatty acid profiles, and wool parameters of growing lambs. J. Anim. Sci..

[9] Urrutia O., Mendizabal J.A., Insausti K., Soret B., Purroy A., Arana A. (2016). Effects of addition of linseed and marine algae to the diet on adipose tissue development, fatty acid profile, lipogenic gene expression, and meat quality in lambs. PLoS ONE.

[10] Díaz M.T., Pérez C., Sánchez C., Lauzurica S., Cañeque V., González C., De la Fuente J. (2017). Feeding microalgae increases omega 3 fatty acids of fat deposits and muscles in light lambs. J. Food Comp. Anal..

[11] Andrés S., Morán L., Aldai N., Tejido M.L., Prieto N., Bodas R., Giráldez F.J. (2014). Effects of linseed and quercetin added to the diet of fattening lambs on the fatty acid profile and lipid antioxidant status of meat samples. Meat Sci..

[12] Nguyen D.V., Flakemore A.R., Otto J., Ives S.W., Smith R.W., Nichols P.D., Malau-Aduli A.E.O. (2017). Nutritional value and sensory characteristics of meat eating quality of Australian prime lambs supplemented with pelleted canola and flaxseed oils: Fatty acid profiles of muscle and adipose tissues. Intern. Med. Rev..

[13] Bessa R.J.B., Alves S.P., Santos-Silva J. (2015). Constraints and potentials for the nutritional modulation of the fatty acid composition of ruminant meat. Eur. J. Lipid Sci. Technol..

[14] Ørskov E.R., Benzie D. (1969). Studies on the oesophageal groove reflex in sheep and on the potential use of the groove to prevent the fermentation of food in the rumen. Br. J. Nutr..

[15] Dobarganes García C., Pérez Hernández M., Cantalapiedra G., Salas J.M., Merino J.A. (2005). Bypassing the rumen in dairy ewes: The reticular groove reflex vs. calcium soap of olive fatty acids. J. Dairy Sci..

[16] Martínez Marín A.L., Gómez-Cortés P., Carrión Pardo D., Núñez Sánchez N., Gómez Castro G., Juárez M., Pérez Alba L., Pérez Hernández M., De la Fuente M.A. (2013). Feeding linseed oil to dairy goats with competent reticular groove reflex greatly increases n-3 fatty acids in milk fat. J. Dairy Sci..

[17] Lawlor M.J., Hopkins S.P., Kealy J.K. (1971). The functioning of the oesophageal groove reflex and comparison of the performance of lambs fed individually or in groups. Br. J. Nutr..

[18] Saini R.K., Keum Y.S. (2018). Omega-3 and omega-6 polyunsaturated fatty acids: Dietary sources, metabolism, and significance—A review. Life Sci..

[19] Hopkins D.L., Clayton E.H., Lamb T.A., Van de Ven R.J., Refshauge G., Kerr M.J., Bailes K., Lewandoski P., Ponnampalam E.N. (2014). The impact of supplementing lambs with algae on growth, meat traits and oxidative status. Meat Sci..

[20] Ponnampalam E.N., Burnett V.F., Norng S., Hopkins D.L., Plozza T., Jacobs J.L. (2016). Muscle antioxidant (vitamin E) and major fatty acid groups, lipid oxidation and retail colour of meat from lambs fed a roughage based diet with flaxseed or algae. Meat Sci..

[21] Bligh E.G., Dyer W.J. (1959). A rapid method of total lipid extraction and purification. Can. J. Biochem. Physiol..

[22] Santercole V., Mazzette R., De Santis E.P.L., Banni S., Goonewardene L., Kramer J.K.G. (2007). Total Lipids of Sarda sheep meat that include the fatty acid and alkenyl composition and the CLA and *trans*-18:1 isomers. Lipids.

[23] Esquivelzeta C., Casellas J., Fina M., Campo M.D.M., Piedrafita J. (2017). Carcass traits and meat fatty acid composition in Mediterranean light lambs. Can. J. Anim. Sci..

[24] Gómez-Cortés P., Galisteo O.O., Avilés Ramírez C., Peña Blanco F., de la Fuente M.A., Núñez Sánchez N., Martínez Marín A.L. (2019). Intramuscular fatty acid profile of feedlot lambs fed concentrates with alternative ingredients. Anim. Prod. Sci..

[25] Pérez-Palacios T., Ruiz J., Martín D., Muriel E., Antequera T. (2008). Comparison of different methods for total lipid quantification in meat and meat products. Food Chem..

[26] Bond L.M., Miyazaki M., O’Neill L.M., Ding F., Ntambi J.M., Ridgway N.D., McLeod R.S. (2016). Fatty acid desaturation and elongation in mammals. Biochemistry of Lipids Lipoproteins and Membranes.

[27] Ferreira E.M., Pires A.V., Susin I., Gentil R.S., Parente M.O.M., Nolli C.P., Meneghini R.C.M., Mendes C.Q., Ribeiro C.V.D.M. (2014). Growth, feed intake, carcass characteristics, and meat fatty acid profile of lambs fed soybean oil partially replaced by fish oil blend. Anim. Feed Sci. Technol..

[28] Gómez-Cortés P., Juárez M., de la Fuente M.A. (2018). Milk fatty acids and potential health benefits: An updated vision. Trends Food Sci. Technol..

[29] Costa M., Alves S.P., Francisco A., Almeida J., Alfaia C.M., Martins S.V., Prates J.A.M., Santos-Silva J., Doran O., Bessa R.J.B. (2017). The reduction of starch in finishing diets supplemented with oil does not prevent the accumulation of *trans*-10 18: 1 in lamb meat. J. Anim. Sci..

[30] Santos-Silva J., Francisco A., Alves S.P., Portugal P., Dentinho T., Almeida J., Soldado D., Jerónimo E., Bessa R.J.B. (2019). Effect of dietary neutral detergent fibre source on lambs growth, meat quality and biohydrogenation intermediates. Meat Sci..

[31] Bessa R.J.B., Alves S.P., Jerónimo E., Alfaia C.M., Prates J.A., Santos-Silva J. (2007). Effect of lipid supplements on ruminal biohydrogenation intermediates and muscle fatty acids in lambs. Eur. J. Lipid Sci. Technol..

[32] Altomonte I., Salaria F., Licitra R., Martini M. (2018). Use of microalgae in ruminant nutrition and implications on milk quality-A review. Livest. Sci..

[33] Cooper S.L., Sinclair L.A., Wilkinson R.G., Hallett K.G., Enser M., Wood J.D. (2004). Manipulation of the n-3 polyunsaturated fatty acid content of muscle and adipose tissue in lambs. J. Anim. Sci..

[34] Alvarenga T.I.R.C., Chen Y.Z., Lewandowski P., Ponnampalam E.N., Sadiq S., Clayton E.H., Van de Ven R.J., Perez J.R.O., Hopkins D.L. (2016). The expression of genes encoding enzymes regulating fat metabolism is affected by maternal nutrition when lambs are fed algae high in omega-3. Livest. Sci..

[35] EFSA (2012). Scientific opinion on the tolerable upper intake level of eicosapentaenoic acid (EPA), docosahexaenoic acid (DHA) and docosapentaenoic acid (DPA). EFSA Panel on Dietetic Products, Nutrition and Allergies (NDA). EFSA J..

[36] EU (2010). Amending Regulation (EC) No 1924/2006 of the European Parliament and of the Council with regard to the list of nutrition claims. Commission Regulation (EU) No 116/2010 of 10 February 2010. Off. J. Eur. Union.

[37] Alvarenga T.I.R.C., Chen Y., Furusho-Garcia I.F., Pérez J.R.O., Hopkins D.L. (2015). Manipulation of omega-3 PUFAs in lamb: Phenotypic and genotypic views. Compr. Rev. Food Sci. Food Saf..

[38] Drouin G., Rioux V., Legrand P. (2019). The n-3 docosapentaenoic acid (DPA): A new player in the n-3 long chain polyunsaturated fatty acid family. Biochimie.

[39] Nguyen D.V., Malau-Aduli B.S., Cavalieri J., Nichols P.D., Malau-Aduli A.E.O. (2018). Supplementation with plant-derived oils rich in omega-3 polyunsaturated fatty acids for lamb production. Vet. Anim. Sci..

[40] Simopoulos A.P., Alvergne A., Jenkinson C., Faurie C. (2016). Evolutionary aspects of the dietary omega-6/omega-3 fatty acid ratio: Medical implications. Evolutionary Thinking in Medicine. Advances in the Evolutionary Analysis of Human Behaviour.

[41] Chen J., Liu H. (2020). Nutritional indices for assessing fatty acids: A Mini-Review. Int. J. Mol. Sci..

